# Mobile App for Pre- and Intraoperative Assessment of Astigmatism Correction with Toric Intraocular Lenses in Cataract Surgery

**DOI:** 10.1155/2020/8354140

**Published:** 2020-03-15

**Authors:** Francisco Aecio Fernandes Dias, Vinicius Jose Fernandes Dias, Barbara de Araujo Lima Dutra, Anna Christina Siqueira Marques, Edgar Marçal, Abrahao da Rocha Lucena, Joao Crispim Ribeiro

**Affiliations:** ^1^Department of Ophthalmology, Christus University Center (Unichristus), Fortaleza, Brazil; ^2^Ceara Vision Institute, Fortaleza, Brazil; ^3^Institute Virtual University, Federal University of Ceara, Fortaleza, Brazil

## Abstract

**Purpose:**

To develop a mobile app that allows capturing and editing of photographs, performs image transposition and projection of a protractor with 360° axis markings, and permits accurate visualization of programmed alignment for the positioning of toric intraocular lenses (IOLs).

**Methods:**

In this prospective case series study, a codesign methodology was chosen to develop the Eye Axis Check application. After app development, measurements were obtained and comparisons were made between manual marks and toric IOL alignment without and with the app in 30 eyes that had undergone cataract surgery with toric IOLs. The mobile app was made available to 15 ophthalmic surgeons in different cities to assess its usability.

**Results:**

The users approved the developed application for its ease of use and utility. The mean difference between the markings made manually and those made with the app was 1° (±2°; range: 0°–5°), and the mean difference between the IOL position and the assessment made by the app was 3° (±3°; range: 0°–12°). Upon comparison of the agreement between the app measurements and the manual measurements for the IOL angle, no significant differences were found, and an excellent concordance (0.997) and a strong positive linear correlation (0.995) were observed.

**Conclusion:**

A mobile app for preoperative planning and intraoperative toric IOL alignment was developed and revealed to be useful and easy to use.

## 1. Introduction

Treatment of corneal astigmatism is an important factor in cataract surgery, since residual astigmatism may compromise a patient's uncorrected visual acuity after surgery. It has been estimated that 30% of patients with cataracts have more than 0.75 D of corneal astigmatism, that 22% have more than 1.50 D, and that 8% have more than 2.00 D [1]. Toric intraocular lenses (IOLs) are used to correct corneal astigmatism that coexists in patients undergoing cataract surgery. Accurate alignment of an IOL is important for its effectiveness in astigmatism correction. Inappropriate intraocular lens positioning may be due to intraoperative alignment error or postoperative IOL rotation [2]. Thus, the IOL must be positioned on the exact axis or meridian previously selected based on preoperative exams. It is known that, for every 3 degrees of deviation from the predetermined axis, the IOL loses 10% of its astigmatism correction power. A rotation of 30 degrees from the programmed axis can result in the loss of 100% of the astigmatism correction. Therefore, intraoperative confirmation of markings made to correctly position IOLs is a determining factor in the refractive outcome of the surgery [3].

Ophthalmic applications are making the smartphone a medical tool, and there are more than 342 eye-care applications available. From 2009 to 2012, there was a 9-fold increase in the number of surgical applications available on smartphones [4].

This study aimed to develop a mobile app to verify the marking of eyes planned for cataract surgeries with toric IOLs and to aid in the visualization of correct IOL alignment.

## 2. Methods

### 2.1. Study Design

The study was carried out in accordance with the protocol number 62660116.3.0000.5049, approved by the Research Ethics Committee of Christus University Center (Unichristus), and the ethical principles that govern the Declaration of Helsinki.

The work was divided into three stages: development and creation of the mobile app, realization of the study with a series of surgical cases in which the application was used and the results were documented and analyzed, and evaluation of the usability of the application as indicated by different surgeons in their responses to a validated questionnaire.

### 2.2. Mobile App

The measurement process is based on a previously taken reference photograph of the eye at the 0–180° position and on subsequent comparison of the implant. The positioning of the photograph is guided by a bubble-level system that uses the inclinometer available on the hardware of the smartphone to obtain a properly aligned image. Even so, there is the option to make subsequent adjustments in the markings by making use of a line that can be moved and rotated in the *XY* axis. It is even possible to take a new photograph of the eye to see if the implants are correctly positioned.

Clicking on the IOL Module option during phacoemulsification with toric IOL implants takes the user to a screen with three new keys: “Surgical Planning,” “IOL Position Photographing,” and “IOL Axis Analysis”; in addition, a tutorial is available through the “Tutorial” key in the upper right corner.

**Figure 1:**
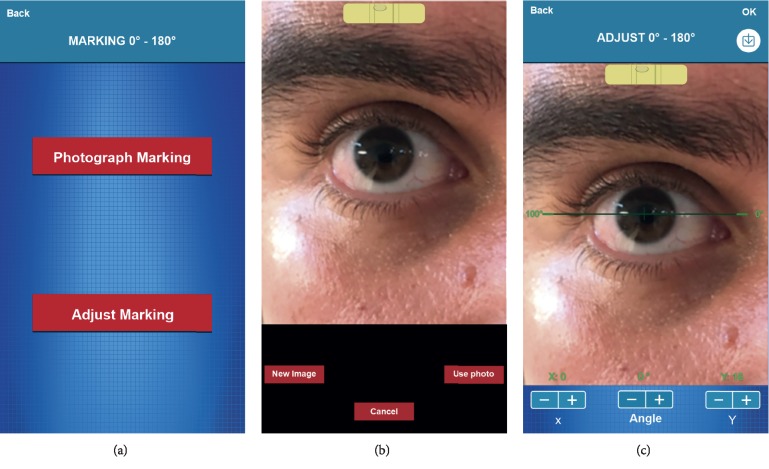
(a) “Marking 0°–180°,” (b) “Photograph Marking,” and (c) “Adjust Marking” screens from the app. It is possible to verify whether there is any tilt from the 0°–180° marking and to adjust if needed.

Clicking on the Marking 0°–180° key opens the next screen, which allows the user to photograph the 0°–180° markings already made in the patient's eye with a manual marker (pendulum, bubble level, electronic marker, etc.). When selecting “Photograph Marking,” the user should be positioned in front of the patient with the 0°–180° marking already made in the eye. After the patient's eye has been photographed with the 0°–180° markings, the “Adjust Marking” screen allows the user to adjust the photograph, so it overlays the markings made in the patient's eye (Figure 1). The application would be useful to assess whether the manual dialing is really not in the correct location. In our study, all eyes were subjected to this comparison. At the end of the surgery, it is still possible for an assistant to take a photo of the monitor where the surgery is being transmitted to verify that the implant is correctly positioned.

**Figure 2:**
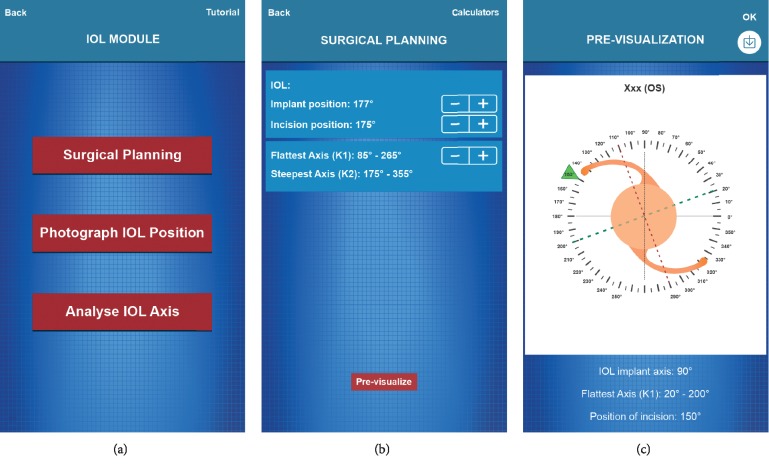
(a) Screen for access to the surgical planning and tutorial screens. (b) Surgical Planning screen. (c) Pre-visualization of surgical planning. The user can record the image for later overlay of images at the end of the procedure, providing a way to assess the measurements.

The Surgical Planning screen is populated with information according to the patient's preoperative exams: the implant position in degrees, the incision position, and the patient's keratometric measurements. The flattest axis (*K*1) and the steepest axis (*K*2) of the cornea are also recorded. In addition, the user can see a simulation of the surgical plan in a graphical format when the proper surgical planning data and patient information are recorded (Figure 2) and can save the image in the application for later overlay of images at the end of the measurement process, providing a record of the measurements.

On the Surgical Planning screen, the user has online access to the toric lens calculators of the four manufacturers of toric IOLs on the market (Alcon, Johnson & Johnson, Rayner, and Zeiss).

**Figure 3:**
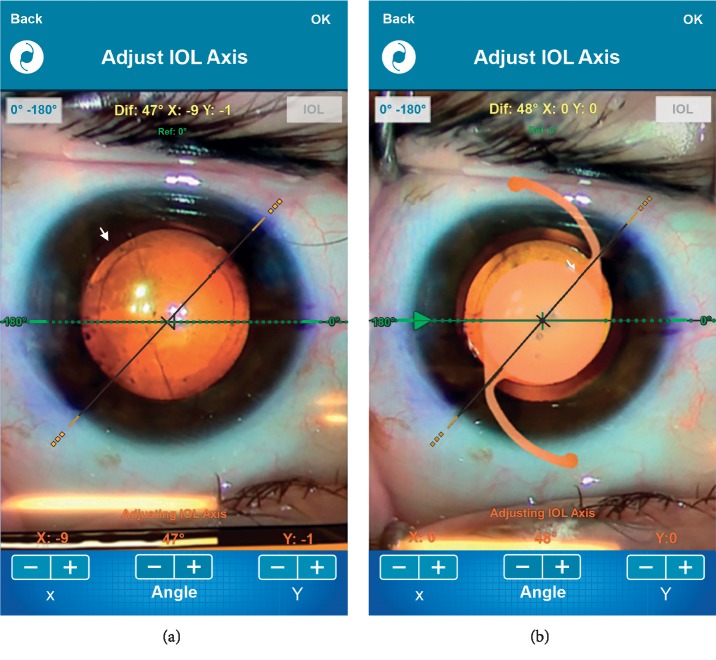
(a) Image from the video system coupled to the surgical microscope to adjust the IOL axis. (b) Overlay of the surgical planning images on the photo from the monitor adjusted to the IOL axis.

When the patient is on the surgical table, a second marking is made that refers to the position of the axis of the lens with a Mendez ring; alternatively, according to the routine of the surgeon, this may have already been done with one of the various manual markers on the market. The surgery is performed, the toric IOL is implanted as planned, and a photograph of the TV monitor coupled to the surgical microscope chamber is acquired by clicking “Photograph IOL Position”; this again accesses the camera of the device, and the mobile protractor and bubble level can be referenced to guarantee the correct alignment of the hand at the moment of the photo (Figure 3).

There is an option to save to the camera roll or print the image. In the upper left corner, there is another small circular key with the shape of an intraocular lens, selection of which allows the user to verify the markings against the surgical plan by overlapping the IOL images, incision positions, and corneal meridians from the surgical planning stage. This step completes the target alignment of the axes for intraocular lens implantation.

IOL angle measurements were performed on 30 eyes. A *t*-test for paired data was used to compare the two methods.

### 2.3. Case Series

Individuals underwent cataract surgery with toric intraocular lens implantation by the same surgeon at the Ceara Vision Institute from March to October of 2018 with the due consent of the institution and after free and informed consent was provided by each participant in the study.

The inclusion criteria included cataract and regular astigmatism of more than 1.25 diopters. Subjects with cataract who had irregular astigmatism or regular astigmatism of less than 1.25 diopters and those who refused to sign the informed consent form were excluded from the study.

The app was used, measurements were obtained, and comparisons were made between the manual marks and toric IOL alignment without and with the app. Manual marking was performed using conventional toric marking tools (a pendulum, a bubble level, or an electronic device).

### 2.4. Pre- and Intraoperative Mobile App Usability

The mobile app was made available to 15 ophthalmologic surgeons in different cities. Through a usability test, the study aimed to determine whether the participants could verify the alignment of the marks before and during cataract surgery with implantation of toric lenses to correct astigmatism.

There are different standardized questionnaires available in the literature to evaluate the responses of participants of usability tests that are capable of assessing the quality of different characteristics of a system. Our questionnaire was a modified form of the Technology Acceptance Model (TAM) implemented by Davis in 1986 [5]. This questionnaire considers users' perceptions about usability and utility as the main factors affecting the level of acceptance of any technology.

In this test, answers to the following questions were determined: if the app for measuring the alignment of markers preoperatively and intraoperatively via mobile phone was viable; if the results obtained with the mobile device were comparable to those obtained with traditional methods; if the markings were unaffected; if the time needed to carry out verification with the app was a limiting condition for its use; if the developed system presented good levels of usability; if there were good levels of usefulness for clinical practice; and what the positive aspects, negative aspects, and suggestions for improvement to the presented app were.

The questionnaire was sent online to participants using the Google Forms template, through which it was possible to access real-time responses.

### 2.5. Statistical Analysis

The measurements of the IOL axis made with the app and by the manual methods were initially analyzed with the Shapiro–Wilk test. With regard to descriptive statistics, the mean and standard deviation were calculated, and nonparametric methods were used for statistical analysis. Comparisons of IOL axis measurements between the app and the manual methods were performed using the Wilcoxon test for paired data. The intraclass concordance coefficient (ICC) and its 95% confidence interval were used to evaluate the agreement between the manual and app axis measurements. The degree and direction of the linear correlation between the manual and application method measurements were, in turn, evaluated by Spearman correlation coefficient (*r*) analysis. The agreement between the manual and the app method measurements was also evaluated by the Bland–Altman graphical method.

In all analyses, a significance level of 5% was considered to indicate statistical significance (a *p* value less than 0.05). GraphPad Prism software version 6.00 for Mac (GraphPad Software, San Diego, California, USA) was used to implement statistical procedures and for graphing.

## 3. Results

All patients underwent both methods of marking. There were no statistically significant differences between the markup performed by the application and the manual method (Table 1).

The degree of agreement between the measurements obtained by the two methods was evaluated by the intraclass correlation coefficient (ICC). The ICC values showed excellent agreement between the measurements of the IOL angle (ICC = 0.997; IC: 0.995 to 0.999; *P* < 0.001). The ICC calculation was based on a two-factor model in which both factors were random: the 30 participating cases for IOL implantation and the 2 measurement methods (random two-factor model).

The degree and direction of the linear correlation between the measurements obtained by the two methods were measured by Spearman correlation coefficient analysis. The Spearman coefficient (*r*) values showed a strong positive linear correlation between the measurements obtained by the two methods for the toric IOL angle (*r* = 0.995; *P* < 0.0001).

**Figure 4:**
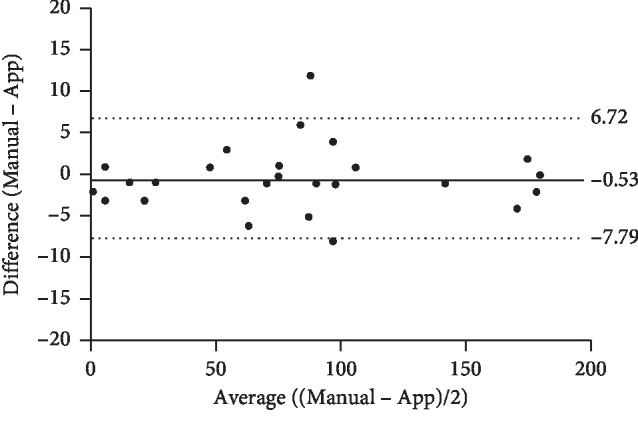
Evaluation of the agreement between the measurements of the IOL angle made by the application and those made by the manual method in 30 eyes with a Bland–Altman graph.

Regarding concordance evaluation using a Bland–Altman graph, the differences between the IOL angle measurements obtained by the application and by the manual method in 30 eyes were homogeneously distributed along the horizontal axis and were concentrated around the mean, except for a point that exceeded the limits of agreement. The mean value of the differences was very small, being equal to −0.53 degrees, with a standard deviation of 3.70 degrees. In addition, the limits of agreement defined a narrow range (−7.79 to 6.72 degrees). These findings indicated that the agreement between the measurements obtained by the two methods was very good (Figure 4).

Thirteen of 15 colleagues who tested the Eye Axis Check app answered the usability questionnaire. Two colleagues reported that they had not had the opportunity to perform toric IOL implantation in a timely manner.

The questions evaluated the utility of the application as perceived by the participants, and the answers are summarized in Table 2. According to the provided answers, 100% of the users of the application agreed that it allowed them to document measurements, quantify misalignment, and identify faults in the markings. In total, 92.3% felt that the application was useful for making repositioning decisions and for planning the surgery, and 84.6% of users found the application to be useful in IOL repositioning.

## 4. Discussion

A meta-analysis in 2016 of 13 randomized clinical trials concluded that toric lenses were more effective in reducing astigmatism than monofocal lenses in patients who underwent cataract surgery with or without the use of intraoperative relaxing incisions [6].

Inaccuracy of measurements of the eye obtained during preoperative examinations, inaccuracy of the orientation of the intraocular lens relative to the desired or calculated target and, to a lesser extent, rotation of the intraocular lens postoperatively are the main causes for the appearance of residual astigmatism [7]. If the rotation of lenses with the new platform is insignificant, the critical steps are alignment of the patient with accuracy in preoperative exams to determine the steepest intraoperative meridian and alignment of the toric lens once this meridian has been determined.

Toric lenses have different optical powers for different meridians, so they must be correctly aligned to neutralize corneal astigmatism [8]. There are several tools for marking the desired target or axis to align an IOL, from manual markers of various types and models to digitally guided imaging systems, which were found to be more accurate in a prospective study on 60 eyes of 60 patients [2].

In a prospective randomized study, 22 participants were randomly allocated into two groups to compare the methods for toric intraocular lens marking (both free-hand and slit-lamp), and subsequently, the values obtained were compared to those obtained with the toriCAM application. The mean absolute error of all markings prior to toriCAM adjustment was 3.18° ± 2.22°. This was reduced to 1.28° ± 1.34° after use of the application (*P* < 0.001). The toriCAM application for smartphones could improve the accuracy of preoperative marking methods for toric intraocular lens alignment [9]. Another application (Goniotrans) can aid in postoperative measurement of toric axes with camera-enabled cell phones [10]. These applications are different from our application because our tool is capable of measuring the axes before surgery, facilitating correct implantation of the IOL.

The Eye Axis Check application was developed to address the increasing need to improve the orientation of toric intraocular lenses to reduce residual postoperative astigmatism. This application will be completed in a timely manner to be released on the Apple IOS platform and will be available in the Apple Store; the software is registered at the Brazilian National Institute of Industrial Property (INPI) under protocol number BR 512017001573-2 (Eye Axis Check).

However, this study has some limitations. In the initial model of the proposed app, some features could have made the application more complete. For example, the lack of ability to store images for successive patients from the same surgical day and the lack of ability to connect the application to another device that could do so forced the users to delete each case before adding another. This deficiency has been mitigated by the possibility of saving the images to the camera roll of the smartphone or printing them. Nevertheless, the scope of the application, enabling access to the calculators of the main intraocular lens manufacturers, and the diversity of language options in a single application, including Portuguese, English, and Spanish, enrich and will tend to broaden interest in the application. In addition, it is possible to create a surgical plan for a procedure; the plans can be printed, taken to the surgical center, and overlaid at the end of the measurement, enabling assessment of the result. Similar to the case in the tests carried out by the developer, the literature has shown misalignment of toric lens axis markers created with manual markers during cataract surgery [11].

In the case series, the data obtained by the app presented a minimal standard deviation from the target axis of the intraocular lenses relative to the manual 0°–180° reference mark. When the Wilcoxon test for paired data was applied, no single set of data presented a statistically significant difference between the two methods. However, the findings had important clinical relevance, since 3 degrees of IOL deviation cause an approximate loss of 10% of astigmatism correction [3]. Moreover, with an increase in astigmatism, the impact of a change in the position of the eye due to cyclotorsion on the correction is greater [12].

The Spearman coefficient (*r*) values revealed a strong positive linear correlation between the measurements obtained by the two methods for the IOL angle. The degree of agreement measured by the Bland–Altman graphical method between the markings made with the application and the manual method with respect to the IOL angle were very well correlated.

Through a usability test, it was finally demonstrated that the participants of the study could verify the alignment of their markings preoperatively and intraoperatively in the context of cataract surgery with toric IOL implantation, indicating that the application is useful in phacoemulsification surgeries with toric IOL implants.

## 5. Conclusion

Eye Axis Check, a mobile app for preoperative planning and intraoperative toric IOL alignment, was developed and revealed to be useful and easy to use. The usability test demonstrated good acceptance of the device by different surgeons participating in this study. Errors in both the 0°–180° marking and the IOL angle, when present, were detected by the application. In addition, assessment of the application did not reveal any statistically significant differences between the application-assisted markings and the manual markings relative to the 0°–180° reference marks.

## Figures and Tables

**Table 1:** Comparison between the application and the manual method in relation to the measurements of the IOL angle (target axis).

Angle	Manual method (mean ± SD)	App (mean ± SD)	Mean differences	Meaningfulness (Wilcoxon)
IOL	78.57° ± 51.24°	79.10° ± 50.98°	−0.53	*P*=0.159

SD, standard deviation.

**Table 2:** Summary of responses from part 2 of the usability questionnaire.

Questions	Strongly disagree		Disagree		Indifferent		Agree		Strongly agree	
	*N*°	%	*N*°	%	*N*°	%	*N*°	%	*N*°	%
11. Facilitated the planning of the surgery	—	—	—	—	1	7.7	8	61.5	4	30.8
12. It made it difficult to execute the markings	3	23.1	8	61.5	1	7.7	—	—	1	7.7
13. Permitted to identify markups	—	—	—	—	—	—	8	61.5	5	38.5
14. It complicated the identification of errors	4	30.8	7	53.8	1	7.7	1	7.7	—	—
15. Useful for deciding repositioning	—	—	—	—	1	7.7	7	53.8	5	38.5
16. Useful for repositioning	—	—	—	—	2	15.4	8	61.5	3	23.5
17. It allowed to quantify the misalignment	—	—	—	—	—	—	8	61.5	5	38.5
18. It allowed to document the measurements	—	—	—	—	—	—	9	69.2	4	30.5

## Data Availability

The data used to support the findings of this study are available from the corresponding author upon request. Eye Axis Check application is available for download on the App Store.
